# The *Candida albicans* transcription factor Efg1 governs hyphal morphogenesis independently of the cAMP-protein kinase A pathway

**DOI:** 10.1128/mbio.02913-25

**Published:** 2025-10-31

**Authors:** Juraj Kramara, Rohan S. Wakade, Corey Frazer, Mark A. Stamnes, Richard J. Bennett, Damian J. Krysan

**Affiliations:** 1 Department of Pediatrics, Carver College of Medicine, University of Iowa 4083, Iowa City, Iowa, USA; 2 Department of Molecular Microbiology and Immunology, Brown University 6752, Providence, Rhode Island, USA; 3 Department of Molecular Physiology and Biophysics, Carver College of Medicine, University of Iowa 4083, Iowa City, Iowa, USA; Instituto Carlos Chagas, Curitiba, Brazil

**Keywords:** *Candida albicans*, hyphal morphogenesis, protein kinase A, fungal pathogenesis, transcriptional regulation

## Abstract

*Candida albicans* is one of the most common causes of human fungal disease. An intensively studied *C. albicans* virulence traits is its ability to adopt both yeast and filamentous morphologies. Accordingly, the regulation of the yeast-to-filament transition has been an area of intense study in medical mycology. A long-standing mechanistic paradigm in this field is that the cAMP-protein kinase A pathway phosphorylates a master transcriptional regulator of *C. albicans* filamentation, Efg1, to drive filamentation. This model has been generalized over the years despite the fact that the initial work showed that it applied to only specific *in vitro* filamentation conditions. Here, we reinvestigated the protein kinase A-Efg1 paradigm by generating new strains containing alleles of *EFG1* with the putative protein kinase A phosphorylation site (T208) mutated to either Ala or Glu which blocks or mimics phosphorylation, respectively. We integrated these alleles into *efg1*∆∆ mutants so that they are expressed from the endogenous promoter. We assayed the effect of these Efg1 mutations on (i) *in vitro* filamentation under a wide range of inducing conditions, (ii) biofilm formation, (iii) *in vivo* filamentation, (iv) virulence in a model of disseminated candidiasis, and (v) gene expression during *in vitro* filamentation. Using updated genetic approaches, we found no evidence that blocking or mimicking protein kinase phosphorylation of Efg1 affected its function during filamentation, biofilm formation, or infection. Therefore, additional studies will be required to identify the mechanisms by which Efg1 is regulated during hyphal morphogenesis.

**IMPORTANCE:**

*Candida albicans* is a common human fungal pathogen causing both superficial mucosal and life-threatening invasive disease. The virulence of *C. albicans* is associated with its ability to form filamentous hyphae and pseudohyphae. The regulation of this process is widely attributed to the phosphorylation of a transcription factor Efg1 by the protein kinase A signaling pathway. Since its initial description 25 years ago, this model has informed the design and interpretation of many studies of *C. albicans*. Through the use of updated genetic methods, we have found that protein kinase A-mediated phosphorylation of Efg1 is dispensable for filamentation, biofilm formation, and virulence in an experimental model of candidiasis. Although these data are negative in nature, the centrality of the protein kinase A-Efg1 paradigm to *C. albicans* pathogenesis studies increases their impact and should catalyze new studies to understand how this master regulator of *C. albicans* biology is itself regulated.

## INTRODUCTION

*Candida albicans* is a common cause of human fungal infections, ranging from superficial infections of oral and genitourinary mucosa to invasive infections of the bloodstream and deep organs such as the liver, kidney, and brain (1). The most intensively studied *C. albicans* virulence trait is its ability to transition between yeast and two filamentous morphotypes, pseudohyphae, and hyphae (2). We will refer to filamentation as the general process by which *C. albicans* forms of both pseudohyphae and hyphae; the individual morphotypes will be referred to specifically by name.

In general, strains and mutants that do not form hyphae have reduced virulence in animal models of *C. albicans* infection (3), but this relationship is not absolute (4). All three *C. albicans* morphotypes are present in histological sections of infected tissue, suggesting that each morphotype contributes to pathogenesis. Supporting this notion, studies of mutants in which the transition between yeast and hyphae was controlled exogenously indicate that the yeast phase cells appear to be most important for dissemination or infectivity, while filamentous forms appear to drive tissue damage (5, 6). As with most complex biological processes and phenotypes, exceptions to these general conclusions can be found throughout the literature.

The transcriptional regulation of *C. albicans* filamentation has been a focus of many studies including large-scale systematic analyses of transcription factor mutant libraries using both *in vitro* and *in vivo* model systems (6–8). While a large number of transcription factors affect *C. albicans* filamentation under at least one specific filament-inducing condition, the basic helix-loop-helix transcription factor Efg1 appears to be the one factor that is most consistently required for wild-type (WT) filamentation under a wide range of *in vitro* and *in vivo* conditions and is widely referred to as a master regulator of filamentation (9). Consistent with the complexity of this phenotype alluded to above, under some conditions such as low oxygen or within a solid matrix, an *efg1*∆∆ mutant is hyper-filamentous relative to WT, indicating Efg1 can suppress filamentation (10). *In vivo*, histological sections of oral tissue from gnotobiotic pigs and mice infected with the *efg1*∆∆ mutant show filamentous forms (11). As such, Efg1 is one of the critical regulators of *C. albicans* filamentation for which a number of questions regarding its mechanisms remain to be addressed.

**Fig 1:**
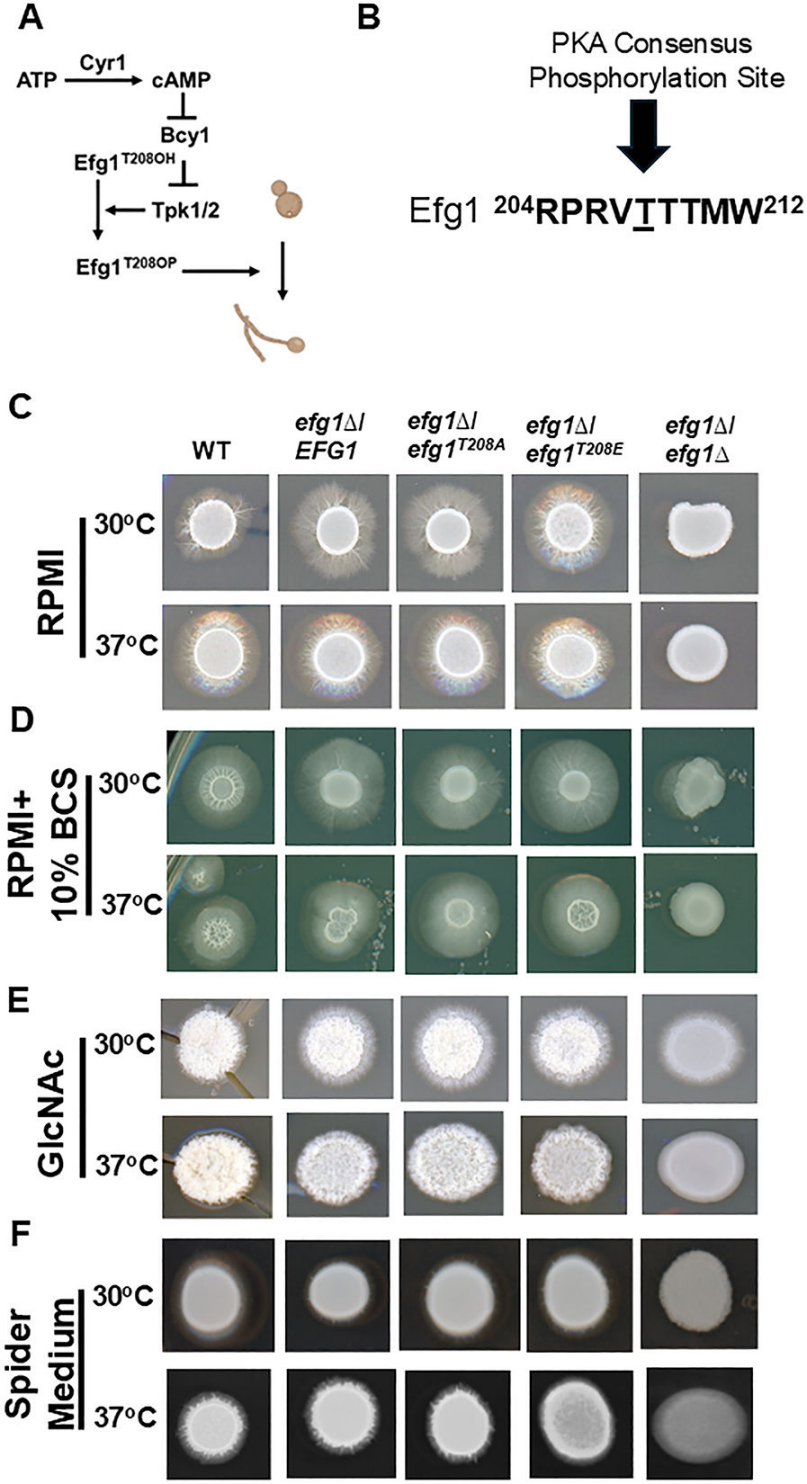
The putative PKA-dependent phosphorylation site threonine-208 does not affect Efg1 function during *in vitro* filamentation on solid agar medium. (**A**) Schematic of PKA phosphorylation dependence of Efg1. (**B**) The putative PKA phosphorylation motif at the threonine 208 position of Efg1 is indicated by a block arrow. (**C–F**) The indicated strains were spotted on agar plates containing the indicated media and incubated at 30°C or 37°C. All strains for a given medium were photographed at the same time point (3–4 days), and phenotypes are representative of two independent isolates.

Among those questions is the mechanism by which Efg1 itself is regulated during filamentation. The standard model for Efg1 regulation, which has informed many studies of *C. albicans* filamentation, involves activation of the cAMP-protein kinase A (PKA) pathway which, in turn, phosphorylates and positively regulates the transcription factor Efg1 (Fig. 1A) (2, 9). Like Efg1, mutants lacking components of the PKA pathway have reduced filamentation under a wide range of *in vitro* inducing conditions (3). The PKA pathway has two catalytic subunits, Tpk1 and Tpk2. The *tpk1*∆∆ mutant shows reduced filamentation under conditions tested to date, while the Tpk2 subunit has a minor effect on filamentation (12, 13). For many years, the PKA pathway was thought to be essential in *C. albicans*, but the *tpk1*∆∆ *tpk2*∆∆ mutant has been constructed by three labs and is not able to filament *in vitro* (14–16).

The mechanistic connection between the PKA pathway and Efg1 is based on three genetic experiments reported by the Ernst lab. First, mutation of Efg1^T208^ (previously reported as T206 in reference 5), a consensus PKA phosphorylation site (Fig. 1B), to alanine reduced filamentation in some inducing conditions (GlcNAc but not yeast peptone dextrose [YPD] supplemented with serum [12]). Second, mutation of the same site to the phospho-mimetic residue glutamate (*efg1*^T208E^) increased filamentation (12). Third, overexpression of *EFG1* in a *tpk2*∆∆ mutant partially restored filamentation, while overexpression of *TPK2* in an *efg1*∆∆ mutant did not (13). Despite the condition-specific effects of the *efg1* mutants, PKA phosphorylation and activation have become the general model for the regulation of Efg1 during hyphal morphogenesis (1).

Using an *in vivo* model of *C. albicans* hyphal induction, we found that deletion of neither the adenyl cyclase gene, *CYR1* (Fig. 1A), nor both PKA catalytic subunits, *TPK1* and *TPK2*, prevented filament in infected mammalian tissue while Efg1 is essential for filamentation under those conditions (15). This discordance between the *in vivo* and *in vitro* roles for PKA and Efg1 could be a more dramatic example of the condition dependence of this relationship initially reported by Ernst (12). However, the generality of the PKA-Efg1 model has also been called into question by other observations reported in the literature. First, the isolation of by-pass suppressors of the PKA pathway by the Konopka lab demonstrated that cAMP-independent mechanisms can mediate *C. albicans* filamentation under some conditions (17, 18). Second, the Konopka (18), Wang (14), and Hogan (19) labs have reported phosphoproteomic analyses of *C. albicans* phosphorylation during filamentation under different conditions showing that Efg1 is a phosphoprotein but finding no evidence of phosphorylation at Efg1^T208^ or at any other potential PKA substrate motif. Based on these observations, we revisited the relationship between the PKA pathway and Efg1 *in vitro* by characterizing the phenotypes and transcriptional effects of *efg1^T208^* mutants generated by re-integration of the mutant alleles at the chromosomal locus under the native *EFG1* promoter.

## RESULTS

### The *efg1^T208A^* mutation does not affect *C. albicans* filamentation or biofilm formation *in vitro*

The original genetic system used by Ernst and co-workers to investigate the role of the putative PKA phosphosites in Efg1 function relied on condition-dependent expression of each allele from the inducible *PCK1* promoter as part of a construct integrated at an ectopic locus relative to the endogenous chromosomal position of *EFG1* (12, 13). Efg1 has a long 5′ untranslated region (UTR) that plays a complex role in regulating both expression of its message and translation of the protein (20). It is unclear how these technical features of the system may have affected the phenotypes of the *efg1* mutants. To reduce potential confounding technical effects, we constructed our strains by integrating *EFG1*, *efg1^T208A^*, and *efg1^T208E^* alleles into the immediate 5′ UTR of an *efg1*∆∆ mutant in the SC5314 derived SN genetic background as previously described (21) to give the corresponding heterozygote strains *EFG1*/*efg1*∆, *efg1^T208A^/efg1∆*, and *efg1^T208E^*/*efg1*∆. In this way, the endogenous 5’UTR and promoter regions of the *EFG1* locus are intact.

Our primary goal was to determine if preventing PKA phosphorylation of Efg1 reduced its function during filamentation. Under some conditions, *EFG1* heterozygotes show haploinsufficiency (22). Therefore, we reasoned that the presence of a single allele *EFG1* is likely to increase the sensitivity of these strains for detection of loss of function phenotypes. It should also be noted that Bockmuhl et al. (12) described the putative PKA phosphosite in Efg1 as T206. As discussed in Brenes et al. (23), this numbering does not match the most up-to-date genome sequence of the SC5314 reference strain. Based on this sequence information, the PKA site is T208 for SC5314-derived strains such as the SN background used in this work. The discrepancy in numbering is because Efg1 constructs generated by Bockmuhl et al. (12) are derived from *C. albicans* strain ATCC 10231. The *EFG1* allele that was cloned from this strain to give plasmid pBI-HAHYD has a two amino acid deletion in the N-terminal region resulting in the designation of the PKA site as T206 (See alignment Fig. S1); the T residue is conserved in SC5314, ATCC 10231, and WO1, but the numberings are distinct due to allelic polymorphisms in the *EFG1* sequence of all three strains.

A wide range of media and conditions have been used to study *C. albicans* filamentation *in vitro* with plating on solid agar plates followed by characterization of colony morphology as one of the most commonly used assays. The filamentation of these strains along with the parental WT (SN250) and the *efg1*∆∆ mutant was compared on solid agar plates containing Roswell Park Memorial Institutue Medium 1640 (RPMI) (Fig. 1C), RPMI + 10% bovine calf serum (BCS; Fig. 1D), GlcNAc (Fig. 1E), and Spider medium (Fig. 1F) at 30°C and 37°C. There was no difference in filamentation between heterozygous strains containing WT *EFG1* or *efg1* mutant alleles under these conditions. The set of conditions tested spans relatively strong induction of filaments (RPMI and RPMI + 10% BCS) to relatively weak induction (Spider medium at 30°C). Under no condition does the phosphomimetic *efg1^T208E^*/*efg1*∆ mutant display increased filamentation. In addition, the filamentation of the heterozygous mutants was clearly more robust than the *efg1*∆∆ mutant; if phosphorylation at Efg1^T208^ contributed to its function, then the *efg1^T208A^*/*efg1*∆ strain should have phenocopied the homozygous deletion strain.

**Fig 2:**
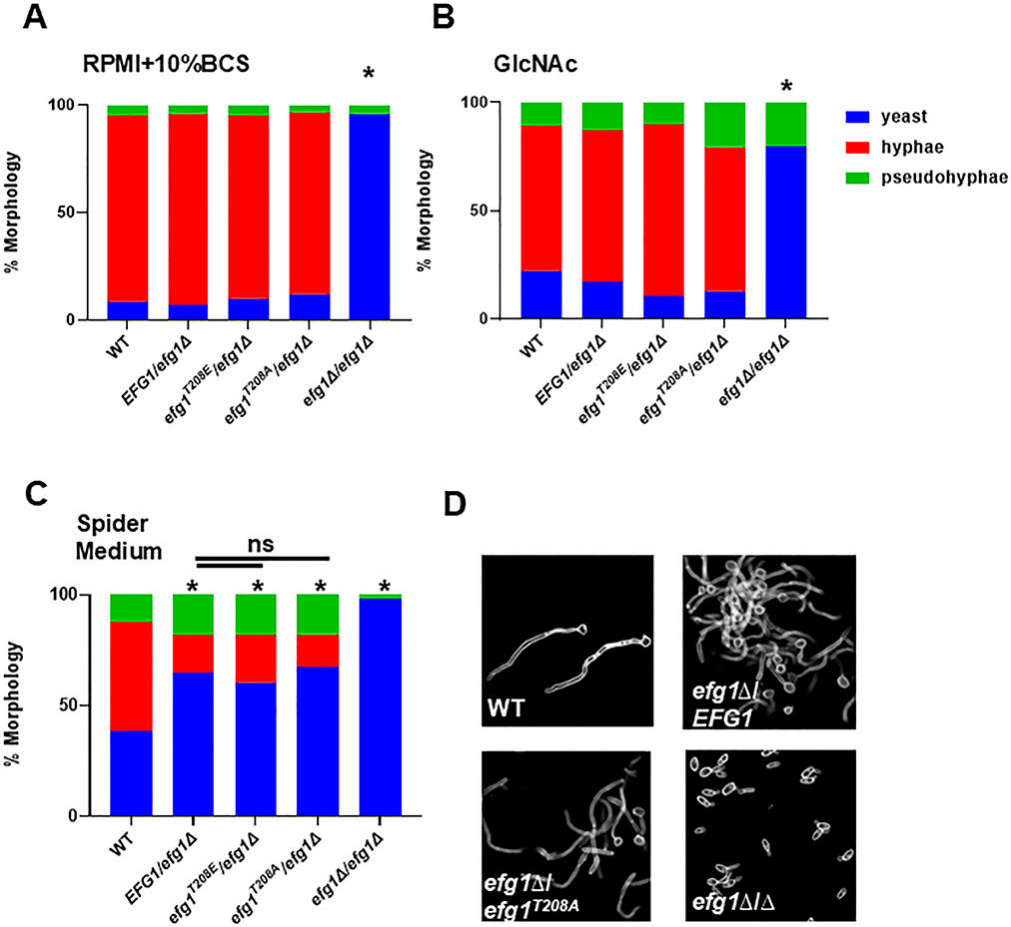
The putative PKA-dependent phosphorylation site threonine-208 does not affect Efg1 function during *in vitro* filamentation in liquid medium. The indicated strains were used to inoculate RPMI + 10% BCS (**A**), GlcNAc (**B**) or Spider medium (**C**) liquid cultures and shifted to 37°C for 4 h. The cells were fixed and the distribution of yeast, pseudohyphae, and hyphae was determined by light microscopy. Bars indicate distributions derived from >100 cells from three independent induction experiments. * indicates statistically significant difference (chi-squared, *P* < 0.05) in distribution relative to WT. (**D**). The photographs show hyphae isolated from the indicated strains induced to form hyphae under GlcNAc medium. The cultures were fixed with formaldehyde (2%), and then cell walls and septa were stained with calcofluor white.

To generate quantitative data, we induced the same set of strains in liquid RPMI + 10% BCS (Fig. 2A), GlcNAc (Fig. 2B), and Spider medium (Fig. 2C). Consistent with the qualitative agar plate assays, no difference between the heterozygous strains containing *EFG1*, *efg1^T208A^*, or *efg1^T208E^* alleles was observed in any of the inducing conditions. Bockmuhl et al. (12) observed the most pronounced reduction in filamentation for the *efg1^T208A^* allele in GlcNAc medium, and our data in Fig. 2B provide a direct comparison. Furthermore, the *efg1*∆/*EFG1* mutant showed haploinsufficiency with Spider medium induction (Fig. 2C) and that phenotype was also observed for both the *efg1^T208A^*/*efg1*∆ and *efg1^T208E^*/*efg1*∆ mutants. Under these conditions, however, we saw no further reduction in filamentation in the *efg1^T208A^*/*efg1*∆ mutant nor increased filamentation with the *efg1^T208E^*/*efg1*∆ mutant. Finally, we stained the cell walls and septa of hyphae isolated from GlcNAc-induced cultures with calcofluor white to assess if any morphological differences were associated with the *efg1^T208A^*/*efg1*∆ mutant. As shown in Fig. 2D, the hyphal length, walls, and septum of the *efg1^T208A^*/*efg1*∆ mutant were indistinguishable from WT and *efg1*∆/*EFG1* cells.

**Fig 3:**
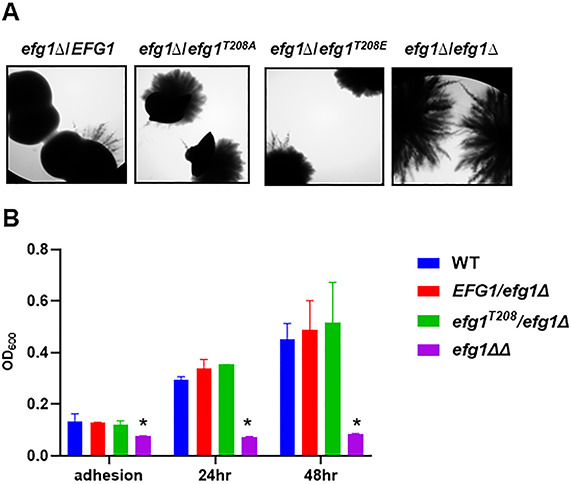
The putative PKA-dependent phosphorylation site threonine-208 does not affect Efg1 function during filamentation in embedded conditions or during biofilm formation. (**A**). The indicated strains were embedded in YP + 2% sucrose medium, top agar plates, and incubated for 5 days at room temperature prior to imaging. (**B**). The indicated strains were inoculated into RPMI + 10% BCS medium for 90 minutes, carefully washed to remove nonadherent cells (adhesion), and incubated for an additional 24 or 48 h in fresh medium. The optical density (OD_600_) of the wells was determined after initial adhesion and then at the two time points as described in Materials and Methods. * indicates statistically significant difference from WT by one-way ANOVA corrected for multiple comparisons (Bonferroni).

In contrast to other conditions, deletion of *EFG1* increases filamentation under embedded conditions (10), and, therefore, if the *efg1^T208A^* mutation reduced Efg1 function, then the *efg1^T208A^*/*efg1*∆ mutant should also increase filamentation when embedded in YPS at ambient temperature. Once again, no evidence of reduced function in the *efg1^T208A^*/*efg1*∆ mutant was evident as the WT and mutant alleles showed similar filamentation, while the *efg1*∆∆ mutant showed the previously reported increase in filamentation (Fig. 3A). Efg1 is also critical for biofilm formation (24) and, therefore, we tested the ability of the *efg1^T208A^*/*EFG1*∆ mutant to form biofilms in RPMI + 10% BCS. As expected, the *efg1*∆∆ mutant was deficient in initial adhesion and had reduced biofilm density at both 24 and 48 h, while the *efg1^T208A^*/*efg1*∆ mutant formed biofilms similar to WT as well as to the *efg1*∆/*EFG1* mutant (Fig. 3B).

### The *efg1^T208A^* mutation does not affect *C. albicans* filamentation *in vivo* and has no effect on virulence in a model of disseminated candidiasis

**Fig 4:**
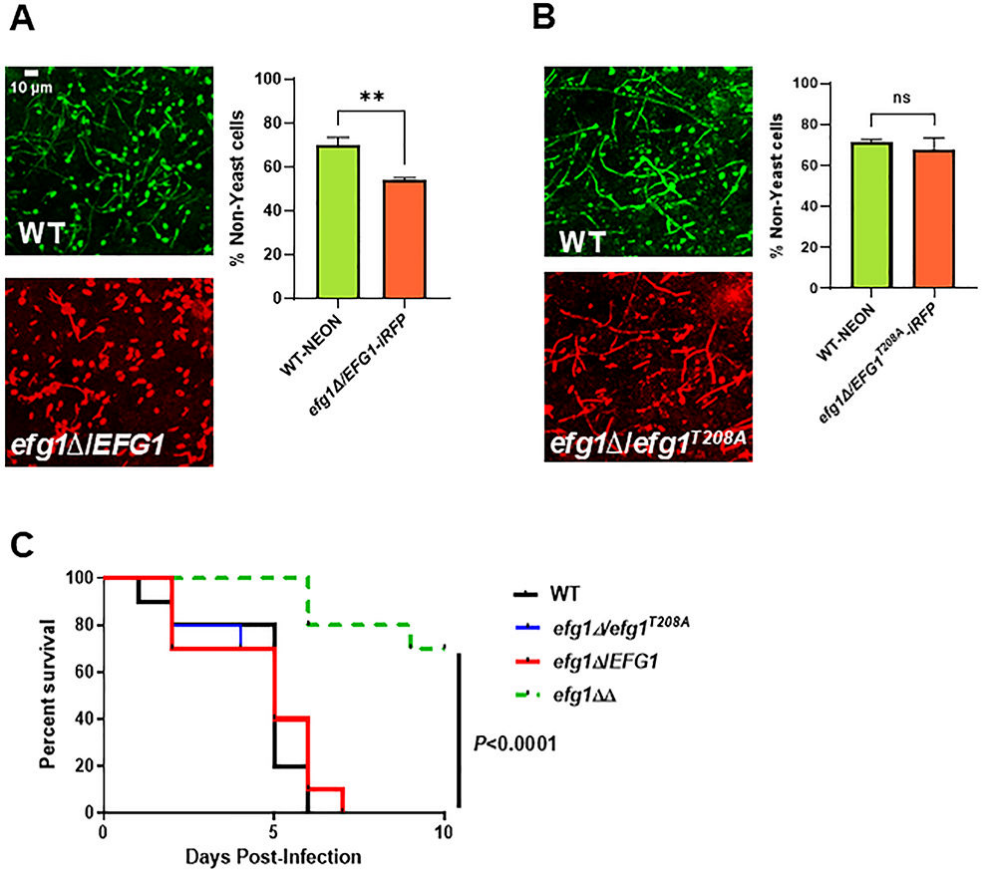
The *efg1^T208A^* mutant is not deficient for *in vivo* filamentation and is virulent in a mouse model of disseminated candidiasis. The *in vivo* filamentation of *efg1*∆/*EFG1* and *efg1T^208A^*/*EFG1* mutants fluorescently labeled with iRFP was compared to WT fluorescently labeled with mNEON as described in Materials and Methods. The *efg1*∆/*EFG1* mutant (**A**) showed modest haploinsufficiency (** indicates *P* = 0.01, Student’s *t*-test, paired), while the *efg1T^208A^*/*EFG1* mutant (**B**) filaments to the same extent as WT in an *in vivo* model of filamentation (NS = non-significant by Student’s *t*-test). The bars indicate percent filamentous cells from >100 cells from multiple fields and two independent infections (NS = non-significant by Student’s *t*-test). (**C**) CD1 mice were infected with the indicated strains (*N* = 10 mice per infecting strain) and monitored for signs of morbidity as described in Materials and Methods. The survival of mice infected with the *efg1*∆/∆ strain was statistically different from all other strains (Kaplan-Meier analysis and log rank (Mantel-Cox test, *P* < 0.005); the WT and *efg1*∆/*EFG1* and *efg1T^208A^*/*EFG1* mutants showed no statistically significant difference.

We have previously shown that *EFG1* and *PKA* pathway mutants have discordant filamentation phenotypes *in vivo* (15). Specifically, the *efg1*∆∆ mutant forms essentially no filaments, while the *tpk1*∆∆ *tpk2*∆∆ mutant as well as the deletion mutant of the adenylyl cyclase enzyme (*cyr1*∆∆) was able to form filaments (15). None of these mutants are able to filament under standard *in vitro* inducing conditions. To further test the role of Efg1^T208^ in this process, we compared the filamentation of the *efg1*∆/*EFG1* and *efg1^T208A^*/*efg1*∆ mutants to WT in our ear model of *in vivo* filamentation (25). In this experiment, a 1:1 mixture of WT cells fluorescently labeled with mNEON and *efg1* mutant orthogonally labeled with iRFP are injected into the sub-dermal tissue of the mouse ear. The filamentation is then quantified as described in Materials and Methods 24 h after infection. The heterozygous WT strain (*efg1*∆/*EFG1*) showed a modest haploinsufficient phenotype (Fig. 4A), while the *efg1^T208A^*/*efg1*∆ mutant was not different from WT (Fig. 4B). It is not clear why the *efg1^T208A^*/*efg1*∆ mutant is slightly more effective at filamentation than the WT allele during *in vivo* filamentation. One possibility is that the mutant allele is expressed at higher levels *in vivo* or that the protein is more stable. The nature of these differences notwithstanding, the *efg1^T208A^*/*efg1*∆ mutant is not deficient relative to the *efg1*∆/*EFG1* mutant, which is consistent with the *in vitro* data and supports our conclusion that phosphorylation of Efg1 at T208 by the PKA pathway does not contribute to its regulation of filamentation *in vivo*.

Both the PKA pathway and Efg1 are required for virulence in a mouse model of disseminated candidiasis (3, 26). It is possible that phosphorylation of Efg1 by PKA may affect virulence through a morphology-independent mechanism. To test that possibility, we infected outbred CD-1 mice with WT as well as the *efg1*∆/*EFG1*, *efg1^T208A^*/*efg1*∆, and *efg1*∆∆ mutants. The mice were monitored for signs of moribundity and sacrificed at that time. As expected, the *efg1*∆∆ mutant is hypovirulent relative to WT (Fig. 4C). However, there was no statistically significant difference in virulence between WT and the *efg1*∆/*EFG1* or *efg1^T208A^*/*efg1*∆ mutant (Fig. 4C). These data indicate that the virulence phenotypes shared by the PKA pathway mutants and the *EFG1* deletion mutant are not directly related through PKA-Efg1 phosphorylation of threonine 208.

### The *efg1*∆/*EFG1* mutant shows a haploinsufficient gene expression profile during filamentation that is not affected by the *efg1^T208A^* mutation

**Fig 5:**
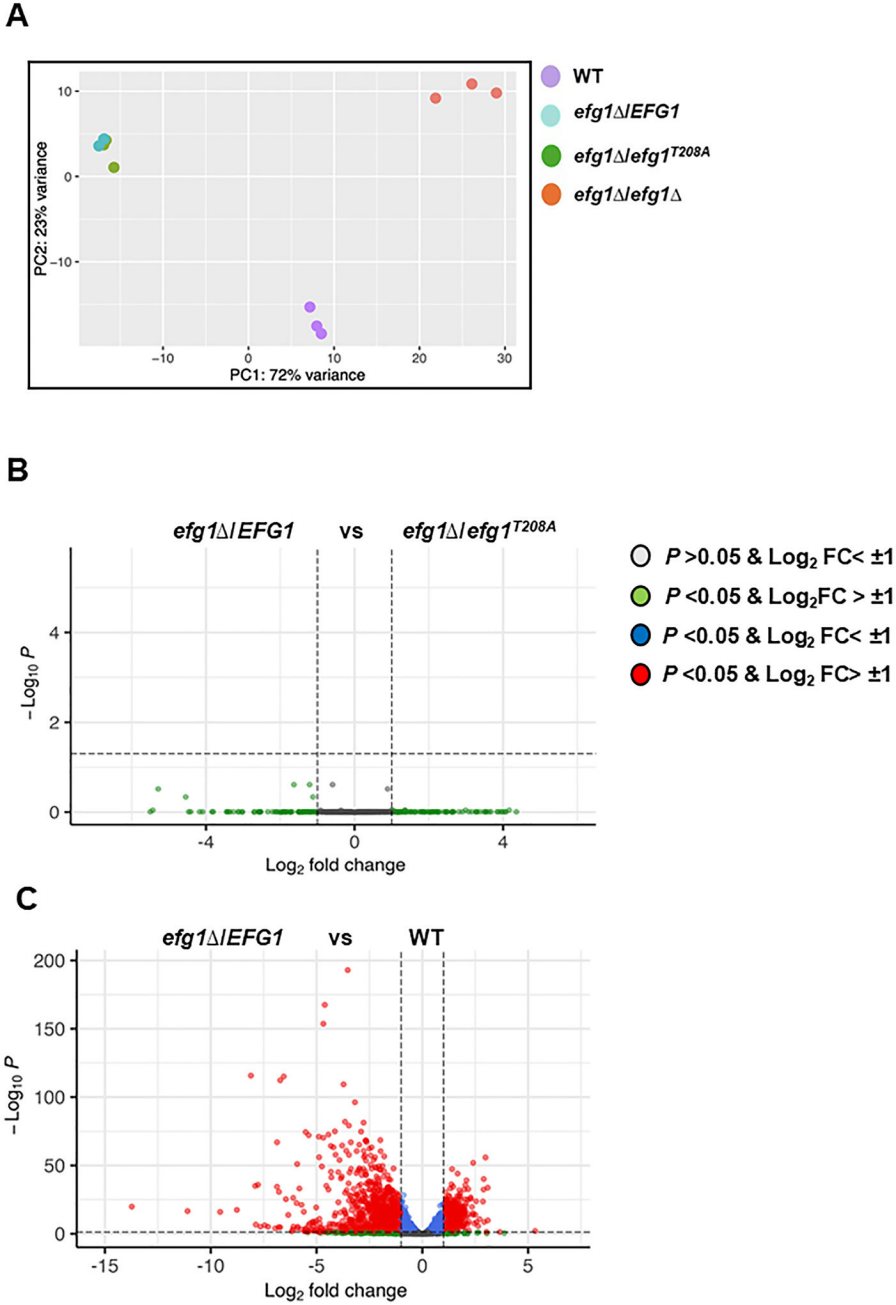
The *efg1^T208A^* mutation does not alter transcriptional function during filamentation *in vitro*. (**A**). PCA of the RNA-seq profiles (raw and processed data: Table S1) for the indicated strains. Each dot indicates a biological replicate (*n* = 3). (**B**) Volcano plot comparing the expression profiles of the *efg1*∆/*EFG1* mutant and the *efg1T^208A^*/*EFG1* mutant. (**C**) Volcano plot comparing WT and the *efg1*∆/∆ mutant. The hatched horizontal lines indicate FDR of 0.05, while the vertical line shows ± 1 log2; the cut-off values for the definition of a differentially expressed gene.

In principle, PKA pathway phosphorylation of Efg1 could affect its transcriptional function without affecting the process of filamentation (27). Therefore, we performed RNA-seq on WT as well as the *efg1*∆/*EFG1*, *efg1^T208A^*/*efg1*∆, and *efg1*∆∆ mutants after 4-h induction in RPMI + 10% BCS (see Table S1 for raw and processed data). Strikingly, principal component analysis (PCA) plots show that the expression profiles for the *efg1*∆/*EFG1* and *efg1^T208A^*/*efg1*∆ mutants closely overlap but, at the same time, are quite distinct from both WT and the *efg1*∆∆ mutants (Fig. 5A). Indeed, there were no differentially expressed genes (log_2_ ± 1; false discovery rate [FDR] < 0.05) between the *efg1*∆/*EFG1* and the *efg1^T208A^*/*efg1*∆ mutants as indicated by the volcano plot in Fig. 5B. Reduced *EFG1* gene dosage in the *efg1*∆/*EFG1* mutant alters the expression of 1,069 genes (426 downregulated; 643 upregulated) relative to WT (Fig. 5C; Table S1). The large effect of reducing the *EFG1* gene dosage by one-half is particularly striking because we did not observe haploinsufficiency during filamentation under RPMI + 10% BCS. The expression of *EFG1* is reduced by ~4-fold in the *efg1*∆/*EFG1* mutant relative to WT under these conditions, which may contribute to the large effect of *EFG1* gene dosage on gene expression. We did not examine the effect of the *efg1^T208A^*/*efg1*∆ mutant on the transcriptional response of *C. albicans* in other inducing medium. These data strongly support the notion that this mutation does not have a significant effect on gene expression during filamentation in this “host-relevant” *in vitro* condition.

## DISCUSSION

Despite the fact that the initial report by the Ernst lab indicated that the PKA-Efg1 phospho-regulatory relationship was limited to specific *in vitro* inducing media (12), this model has been generalized over the years since those data were reported (2, 14). As discussed above, we revisited this relationship because its generality did not seem to match a growing body of data reported in the literature. Our genetic data indicate that under a variety of conditions widely used to induce filamentation *in vitro* as well as during *in vivo* infection, phosphorylation of Efg1 at the putative PKA phospho-site does not affect its function.

The PKA-Efg1 model has informed the design and interpretation of innumerable studies on the mechanisms and regulation of *C. albicans* filamentation and virulence (2, 14). Therefore, the results of our re-visitation of this canonical regulatory relationship using updated genetic approaches have revealed that much is to be learned about how Efg1 is regulated during filamentation. Efg1 is a central regulator of filamentation that affects this process under a wide range of conditions including during infection. Accordingly, the regulation of Efg1 by upstream signaling systems and environmental responses represents a central mechanism driving *C. albicans* pathobiology. Our data indicate that the long-standing paradigm in the field for this regulation is not operative in most of the systems used to study this key *C. albicans* virulence trait. We, therefore, assert that a general model for the regulation of Efg1 function during *C. albicans* filamentation represents a knowledge gap in the field despite its long-standing status as settled science.

At least four reasons for the discordance between our data and the previously reported effects of the *efg1^T208A^* mutation may be operative. First, the effect of genetic background on *C. albicans* phenotypes as part of the larger phenomenon of strain-to-strain heterogeneity is now a well-established source of variation within the field (28). The Ernst lab generated their data using the CAI background which is derived from the reference strain SC5314 (12, 13). Our strains were generated in the SN background which is also derived from SC5314 (29). Second, the initial experiments were performed before the realization that expression of *URA3* could vary dramatically from one heterologous genomic locus to the next (30), and, thus, the *URA3* gene was not expressed from regions of the genome known to support WT levels. Third, the *EFG1* alleles by Ernst were integrated at an ectopic chromosomal locus and expressed from a heterologous, inducible promoter (12). Fourth, the promoter fusions expressed the *EFG1* alleles starting at the ATG codon, not at the transcriptional start site. *EFG1* has a large 5′ UTR that is important for its regulation (20). How these factors might lead to the differences between our data and the previously reported studies is unclear and is not a productive avenue of speculation, in our opinion.

The genetic methods used by Ernst and co-workers (12, 13) were the standard for the field at the time, and it is not our intent to disparage these data or conclusions. It is important, however, to revisit key models when subsequent data suggest a revision may be necessary. Indeed, this is the process by which science moves forward. In this work, we have studied the foundational experiments leading to the PKA-Efg1 regulatory connection using an updated genetic approach and building on the accumulated understanding of Efg1 molecular and cellular biology developed in the literature since those initial studies. The definitive identification of the causal role for phosphorylation of a specific site in a protein is a challenging endeavor. Therefore, it remains possible that our approach is confounded as well in a manner we have not considered. However, a similarly constructed set of *efg1^T208^* mutant strains has been investigated by Brenes et al. (23) in the context of white-opaque switching. They found that the putative PKA phosphorylation site at Efg1^T208^ does affect white-opaque switching, indicating that the PKA-Efg1 mechanism does have biological importance in other settings (23). These findings further support the likelihood that our approach may have found a role for PKA in the regulation of Efg1 during filamentation if it were operative.

In conclusion, the results we have reported, when combined with the previous literature cited above, strongly support the conclusion that PKA regulation of Efg1 is unlikely to be a general mechanism through which morphogenesis-driving stimuli activate this central transcriptional regulator of *C. albicans* filamentation. We hope this work catalyzes additional studies to understand the regulatory mechanisms governing the function of Efg1 in its regulation of this key *C. albicans* virulence trait.

## MATERIALS AND METHODS

### Cultivation conditions and media

Strains were recovered from −80°C storage on YPD agar plates by incubation for 1–2 days at 30°C and used within 2 weeks. Strains were grown overnight in 2 mL YPD cultures prior to all experiments. YPD, Spider medium, and RPMI were prepared using standard recipes (7). To prepare N-acetylglucosamine (GlcNAc) media, glucose in yeast nitrogen base (YNB) minimal media was replaced by 1% (wt/vol) GlcNAc.

### Plasmid and strain construction

The strains were all derived from reference strain SN250 (29) and its corresponding *efg1*∆∆ mutant. The *EFG1* alleles were integrated into the 5′ upstream region of the *efg1*∆∆ mutant by *Hpa1* digestion of *EFG1*^T208^, *EFG1*^T208A^, and *EFG1*^T208E^ constructs cloned into the pSFS2A plasmid followed by standard *C. albicans* transformation methods (21). The presence of the mutant alleles in the resulting heterozygous strains was confirmed by Sanger Sequencing of amplicons generated from the resulting strains. Full details of plasmid construction and the oligonucleotides used are provided in Table S2. The *efg1^T208A^*/*efg1*∆ mutant was labeled with iRFP by transformation with plasmid pENO1-iRFP-NAT1 for use in the *in vivo* filamentation assay. The SN250 mNEON strain was used as a control in those experiments, as has been reported previously (8, 15).

### Agar plate filamentation assays

Overnight cultures of SN250 and *efg1* mutants were diluted to 0.1 OD_600_ and 5 µL was spotted on GlcNAc, Spider medium, RPMI or RPMI + 10% BCS containing agar plates. The strains were incubated at 30°C or 37°C for 3–4 days and photographed. For embedded filamentation assays, the phosphate-buffered saline (PBS)-washed overnight cultures were diluted to ~1,000 cells/mL, and 100–200 μL was plated on YPS media and subsequently overlayed with YPS media. Embedded plates were incubated at room temperature and imaged after days 3–5. The data are representative of at least two replicates.

### Liquid culture filamentation assays

For *in vitro* hyphal induction in liquid media, overnight cultures of the *C. albicans* strains were harvested and diluted 1:50 into liquid media and incubated at 37°C for 4 h. The cells were fixed with 1% (vol/vol) formaldehyde and imaged using the Echo Rebel upright microscope with a 60× objective; pseudohyphae were defined as filamentous cells with non-parallel cell walls, and hyphae were defined as filamentous cells with parallel cell walls. The assays were conducted in biological duplicates on different days. Differences in distribution of morphotypes were analyzed using data pooled from independent replicates and χ^2^ test (*P* < 0.05).

### Biofilm formation assays

Overnight cultures washed twice in PBS, diluted to an OD_600_ of 0.5 in RPMI + 10% BCS. Two-hundred microliters of the suspension was dispensed into 6 wells of a 96-well plate (Corning Incorporated 96-well plate, catalog no. 3596). The cells were then incubated in a 37°C incubator for 90 minutes to allow adherence. Next, the media was removed, and the wells were washed once with PBS to remove non-adhered cells. Adherence was measured OD_600_ using a SpectraMax plate reader. The biofilm-inducing media was replaced, and cells were further incubated at 37°C, without shaking. After 24 or 48 h, the media was aspirated; the cells were washed with PBS as above, and the OD_600_ was measured. Three biological replicates with six technical replicates per strain were analyzed for each strain, and the experiment was repeated independently three times. Differences between strains were analyzed by analysis of variance (ANOVA) and multiple comparison correction with statistical significance set at an adjusted *P* < 0.05.

### *In vivo* filamentation assays

The imaging of dBA/2 mice ears infected with fluorescently mNEON-labeled SN250 and either the iRFP-labeled *efg1*∆/*EFG1* or *efg1^T208A^*/*efg1*∆ mutant was carried out as described previously (8, 15, 25). Each ear was inoculated with a 1:1 mixture of the two strains. Infected ears were imaged 24 h post-inoculation. Multiple Z stacks (minimum 15) were used to score the yeast vs filamentous ratio. The cells were considered yeast if the cells were round and/or budded cells. Furthermore, yeast cells were required not to project through multiple Z stacks. The cells were considered “filamentous” if the cells contained an intact mother cell and filaments that were at least twice the length of the mother body. A minimum of 100 cells from multiple fields were scored. Paired Student’s *t*-test with Welch’s correction (*P* < 0.05) was used to define the statistical significance, which was carried out using GraphPad Prism software.

### Mouse model of disseminated candidiasis

Assessment of the fungal disease progression in a systemic candidiasis model was performed as described previously (11). Female CD-1 outbred mice (10 per group, 6–8 weeks old; Envigo) were inoculated by lateral tail vein injection with 1 × 10^6^ CFU of the indicated *C. albicans* strains, and mice were monitored daily for clinical changes. Mice that demonstrated symptoms of severe diseases such as fur ruffling, difficulty with ambulation, abnormal posture, and/or failure to respond to surroundings were euthanized immediately. Disease progression was analyzed by Kaplan-Meier analysis and log-rank (Mantel-Cox test, *P* < 0.05).

### RNA sequencing analysis of gene expression

The reference strain SN250 and the *efg1*∆/*EFG1*, *efg1*∆/*efg1^T208A^*, and *efg1*∆∆ mutants were subjected to hyphal induction conditions using RPMI supplemented with 10% BCS at 37°C for 4 h. RNA was isolated as previously described (15). Three biological replicates were performed for each strain. Read quality was assessed using FastQC (Babraham Bioinformatics), and then each sample was mapped individually to the *C. albicans* reference genome (SC5314 build 63 from FungiDB) using HISAT2 (1). Raw read counts files were generated, and DESeq2 (2) was used to perform data normalization and differential expression analysis. Differentially expressed genes were defined as those with a ± 1 log2 fold change in expression relative to SN250 and an adjusted *P* < 0.05.

## Data Availability

The raw and processed data have been deposited at the National Center for Biotechnology Information Gene Expression Omnibus website under accession number GSE309112.

## SUPPLEMENTAL MATERIAL

Fig. S1 mbio.02913-25-s0001.tif Alignment of EFG1 sequences used with previous EFG1 sequences.

Supplemental Text File mbio.02913-25-s0002.docx Legends for supplemental material.

Table S1 mbio.02913-25-s0003.xlsx RNA Seq data and analysis files.

Table S2 mbio.02913-25-s0004.xlsx Oligonucleotides and strains used in this work.

ASM does not own the copyrights to Supplemental Material that may be linked to, or accessed through, an article. The authors have granted ASM a non-exclusive, world-wide license to publish the Supplemental Material files. Please contact the corresponding author directly for reuse.
