# Supplementary figures and images for: The Candida albicans transcription factor Efg1 governs hyphal morphogenesis independently of the cAMP-protein kinase A pathway
Source: mBio. 2025 Oct 31;16(12):e02913-25. doi: 10.1128/mbio.02913-25 (PMC12691644; doi:10.1128/mbio.02913-25)

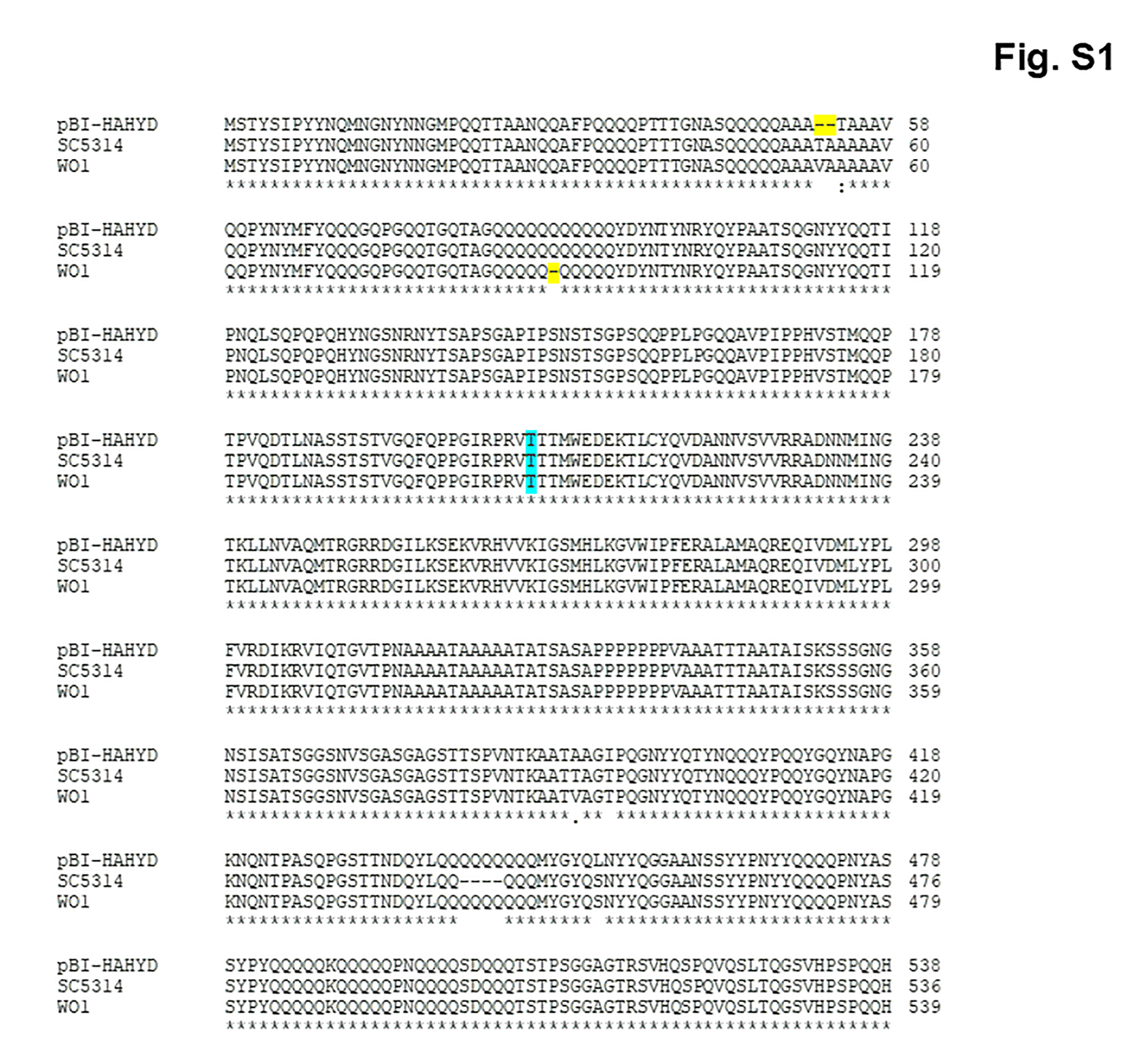

Supplement: Fig. S1 — Alignment of EFG1 sequences used with previous EFG1 sequences. [file mbio.02913-25-s0001.tif]
