# Supplementary material for: The Candida albicans transcription factor Efg1 governs hyphal morphogenesis independently of the cAMP-protein kinase A pathway
Source: mBio. 2025 Oct 31;16(12):e02913-25. doi: 10.1128/mbio.02913-25 (PMC12691644; doi:10.1128/mbio.02913-25)
Supplement: Supplemental Text File — Legends for supplemental material. [file mbio.02913-25-s0002.docx]

**Supplemental Text File**

**Figure S1.** The alignment of *EFG1* sequences from the *EFG1* add back plasmids plasmid used the Ernst lab (pBI-HAHYD, ref. 12) with the two reference strains SC5314 and WO1. Yellow color highlights the deletions in the pBI-HAHYD and WO1 sequences relative to SC5314. Blue shows the position of the putative PKA phosphorylation site.

**Table S1. RNA Seq Data**. RNA sequencing data for WT, *efg1*∆/*EFG1*, efg1^T208A^/*efg1*∆ strains. The first sheet contains a legend for the spread sheets.

**Table S2**. Oligonucleotides and strains.
